# Hypo- and Hyperglycemia Impair Endothelial Cell Actin Alignment and Nitric Oxide Synthase Activation in Response to Shear Stress

**DOI:** 10.1371/journal.pone.0066176

**Published:** 2013-06-12

**Authors:** Steven Frank Kemeny, Dannielle Solomon Figueroa, Alisa Morss Clyne

**Affiliations:** 1 Mechanical Engineering and Mechanics, Drexel University, Philadelphia, Pennsylvania, United States of America; 2 School of Biomedical Engineering, Science and Health Systems, Drexel University, Philadelphia, Pennsylvania, United States of America; 3 Mechanical Engineering and Mechanics, Drexel University, Philadelphia, Pennsylvania, United States of America; University Heart Center Freiburg, Germany

## Abstract

Uncontrolled blood glucose in people with diabetes correlates with endothelial cell dysfunction, which contributes to accelerated atherosclerosis and subsequent myocardial infarction, stroke, and peripheral vascular disease. In vitro, both low and high glucose induce endothelial cell dysfunction; however the effect of altered glucose on endothelial cell fluid flow response has not been studied. This is critical to understanding diabetic cardiovascular disease, since endothelial cell cytoskeletal alignment and nitric oxide release in response to shear stress from flowing blood are atheroprotective. In this study, porcine aortic endothelial cells were cultured in 1, 5.55, and 33 mM D-glucose medium (low, normal, and high glucose) and exposed to 20 dynes/cm^2^ shear stress for up to 24 hours in a parallel plate flow chamber. Actin alignment and endothelial nitric oxide synthase phosphorylation increased with shear stress for cells in normal glucose, but not cells in low and high glucose. Both low and high glucose elevated protein kinase C (PKC) levels; however PKC blockade only restored actin alignment in high glucose cells. Cells in low glucose instead released vascular endothelial growth factor (VEGF), which translocated β-catenin away from the cell membrane and disabled the mechanosensory complex. Blocking VEGF in low glucose restored cell actin alignment in response to shear stress. These data suggest that low and high glucose alter endothelial cell alignment and nitric oxide production in response to shear stress through different mechanisms.

## Introduction

Diabetes affects 8.3% of the United States population (25.8 million people), and an additional 79 million people have pre-diabetes [1]. The majority of diabetic morbidity and mortality relates to cardiovascular disease, and data suggest that the rising diabetes prevalence is increasing the rate of cardiovascular disease [2]. Specifically, people with diabetes suffer from accelerated, severe atherosclerosis, which then leads to heart attack, stroke, and peripheral vascular disease [3], [4]. While diabetes is an established risk factor for atherosclerosis, the mechanism by which diabetes accelerates the disease remains unknown. Glucose fluctuations characteristic of both Type I and Type II diabetes have been implicated in diabetic atherosclerosis, since tight glycemic control reduced the risk of myocardial infarction and stroke in people with diabetes by more than fifty percent [5], [6].

Endothelial cell dysfunction is an initiating step in atherosclerotic plaque development, and altered glucose contributes to endothelial cell dysfunction. Healthy endothelial cells maintain vascular homeostasis through tight control of permeability, inflammation, vascular tone, and injury repair [7]. In contrast, endothelial cells in high glucose are highly permeable, allowing solutes to pass into and through the vascular wall [8]; express increased adhesion molecules [9] and produce less nitric oxide (NO) [10], recruiting more inflammatory cells and reducing vasodilation; and display diminished migration [11] and proliferation [12], thereby inhibiting angiogenesis in response to injury and ischemia [13]. High glucose induces endothelial cell dysfunction via multiple pathways, including mitochondrial superoxide production [14], advanced glycation end-products (AGE) [15], and protein kinase C (PKC) [16]. In a recent study, hypoglycemia similarly elevated endothelial cell mitochondrial superoxide production and decreased NO bioavailability [17].

While atherosclerotic risk factors such as altered blood glucose produce systemic biochemical changes, atherosclerotic plaques primarily develop in regions of disturbed flow. Upon exposure to laminar shear stress, endothelial cells align and organize actin fibers parallel to the flow direction, release NO, and decrease inflammatory adhesion molecules as part of an atheroprotective phenotype [18], [19]. In disturbed flow, which includes low shear stress, flow separation, and flow reversal, endothelial cells are unable to adapt. These cells do not align to the flow and assume an atheroprone phenotype [20]. Altered blood glucose accelerates atherosclerotic plaque development in disturbed flow regions. Some studies further suggest that people with diabetes have diffuse atherosclerotic disease with plaques even in regions of laminar shear stress [21], [22]. We therefore hypothesized that both hyper- and hypoglycemia would inhibit endothelial cell alignment in response to shear stress.

Endothelial cell response to shear stress initiates with deformation of the cell luminal surface, followed by force transmission throughout the cell via the cytoskeleton. Mechanotransduction, the conversion of mechanical force to chemical activity, then occurs at multiple cell locations including cell-cell junctions, cell-matrix adhesions, and the nucleus [23]. Since glucose enhances endothelial cell permeability by disturbing cell-cell junctions, we hypothesized that mechanotransduction would also be diminished or inhibited at these sites [8], [24]. At adherens junctions, flow induces platelet endothelial cell adhesion molecule-1 (PECAM-1) phosphorylation, suggesting that PECAM-1 is the primary mechanosensor [25]. Shear stress also causes vascular endothelial growth factor receptor-2 (VEGFR2) to assemble with adherens junction molecules vascular endothelial cadherin (VE-cadherin) and β-catenin to activate the Akt signaling pathway [26]. A recent study by Tzima *et al* used knockout and transfection models to refine the earlier studies into an endothelial cell adherens junction shear stress mechanosensor that regulates a subset of mechanotransduction pathways [27]. PECAM-1 transmits the mechanical signal via VE-cadherin to VEGFR2, which then activates intracellular signaling via phosphatidylinositol 3-kinase (PI3K). PI3K in turn activates integrins, which activate the Rho-GTPase pathway that eventually leads to actin cytoskeleton reorganization [28], [29]. PI3K further phosphorylates Akt, which is one pathway leading to endothelial nitric oxide synthase (eNOS) phosphorylation and NO release [30]. While PECAM-1 and VE-cadherin knockout cells did not activate VEGFR2 in response to flow, PECAM-1 knockout cells did still align, suggesting that while this mechanosensor is important it is unlikely to be unique.

Several recent papers suggest that elevated glucose inhibits endothelial cell elongation and alignment in response to shear stress due to changes in heparan sulfate proteoglycans in the glycocalyx [31]–[33]. We hypothesized that altered glucose levels may also disturb mechanotransduction via the adherens junction mechanosensor and its associated intracellular pathways. In this study, we examined how both hyperglycemia and hypoglycemia alter endothelial cell alignment and eNOS phosphorylation in response to shear stress as measures of endothelial cell atheroprotective or atheroprone phenotype. High and low glucose effects on cell signaling pathways initiated by the PECAM-1/VE-cadherin/VEGFR2 mechanosensory complex were investigated. This research advances our understanding of how diabetic glucose fluctuations affect endothelial cell response to fluid flow.

## Methods

### Cell Culture, Glucose Conditions, and Shear Stress Exposure

Primary porcine aortic endothelial cells (PAEC, passages 5–9) were isolated from pigs that were euthanized as part of another study (Drexel University and Drexel University College of Medicine Institutional Animal Care and Use Committee approved). PAEC were cultured in Dulbecco’s Modified Eagle’s Medium (DMEM, MediaTech) with 5% FBS, 1% l-glutamine and 1% penicillin-streptomycin (Gibco). pH was maintained during flow experiments by adding HEPES (Sigma) to supplemented medium. Low, normal, and high glucose conditions were defined as 1 mM, 5.55 mM and 33 mM D-glucose and are abbreviated as LG, NG, and HG, respectively. These glucose levels are commonly used to induce glucose-related dysfunction while maintaining cell viability. For LG medium, 1 mM D-glucose was added to DMEM without glucose (Invitrogen). LG medium with 4.5 mM D-mannitol (Sigma) was the LG osmotic control. NG medium was low glucose DMEM (MediaTech). Either D-glucose or D-mannitol was added to low glucose DMEM to a final 33 mM concentration to create HG and HG osmotic control medium, respectively.

For flow studies, a parallel plate flow chamber (Glycotech) enclosed in a 5% CO_2_, 37°C incubator was used to apply 20 dynes/cm^2^ shear stress to PAEC as previously described [34]. Shear stress above 20 dynes/cm^2^ was previously shown to maintain an atheroprotective endothelial cell phenotype [35]. 300,000 cells were seeded in the appropriate glucose medium on collagen Type I coated 25 × 75 mm glass slides (10 µg/mL at 37°C for 3 hours) 48 hours before being assembled into the flow chamber. Shear stress exposure times ranged from 30 seconds to 24 hours, since biochemical signaling occurs within minutes of shear stress whereas morphological changes take 12–24 hours. Shear stress exposure time was shortened to 12 hours for flow experiments in which signaling pathways were dissected with biochemical activators and inhibitors to avoid cytotoxicity. For strain studies, a custom-built apparatus was used to apply cyclic uniaxial strain to cells on elastic membranes as previously described [36]. Briefly, 50,000 cells/cm^2^ were seeded on polydimethylsiloxane (PDMS, Sylgard 184, Dow Corning) membranes with covalently bonded collagen (100 µg/mL) in NG and HG medium for 48 hours. Membranes were then attached to a fixed crossbar at one end and a movable crossbar at the other end. The moveable crossbar was connected to a DC motor via a ball-bearing linear slider (Specialty Motions), which translated motor rotational motion to membrane linear motion. 10% strain at 0.5 Hz was applied for six hours.

### Actin alignment and eNOS Phosphorylation

Endothelial cell shear stress response was assessed by measuring actin alignment and eNOS phosphorylation. For actin alignment, samples were exposed to 24 hours of 20 dynes/cm^2^ shear stress since statistically significant alignment was observed in normal glucose cells at this time point. PAEC were fixed in 4% paraformaldehyde and permeabilized with 0.1% Triton X-100. Actin fibers and nuclei were labeled with rhodamine phalloidin (165 nM, Invitrogen) and bisbenzimide (0.2 µg/mL, Invitrogen), respectively. Samples were imaged at 20x and 60x (minimum resolution 1024×1024 pixels) in an Olympus IX81 inverted confocal microscope.

Actin fiber alignment was analyzed using a custom edge detection Matlab (Mathworks) program [37]. Edge detection uses changes in image intensity to find fiber edges. The maximum intensity gradient direction is then used to calculate the fiber orientation angle. In our edge detection implementation, Sobel operators were applied to grayscale image intensity matrices, and horizontal and vertical convolution values were calculated for each pixel. Pixel edge intensity gradient magnitude and fiber angle were determined from these convolution values. Since edge detection produced fiber angle distributions from −90° to 90°, mean fiber angle was consistently near 0° for all distributions. We therefore used either the aligned fiber percentage (fiber angles between −20° and 20°) or the mean fiber angle for the absolute value angle distributions (0–90°) to determine alignment.

eNOS is phosphorylated in endothelial cells within 30 seconds of shear stress initiation and sustained for up to an hour [30]. We visualized phosphorylated eNOS (p-eNOS) 30 seconds after shear stress initiation by immunofluorescent microscopy to examine activated protein quantity and location. PAEC were fixed in 50∶50 acetone:methanol and incubated with primary antibodies for p-eNOS (pS1179, 1∶100, Invitrogen), followed by the appropriate secondary antibody (Alexa Fluor® 633; Invitrogen) and bisbenzimide. Samples were imaged by confocal microscopy.

### PKC, ROS, and VEGF

Altered glucose levels increase PKC, ROS, and VEGF release. We therefore measured these parameters in LG, NG, and HG cells. Intracellular PKC was detected with Fim-1 diacetate (Santa Cruz), a membrane permeable fluorescein-conjugated bisindoylmaleimide that binds to and inhibits the catalytic PKC domain. PAEC were fixed with 4% paraformaldehyde and permeabilized with 98% methanol. Cells were then incubated with 200 nM Fim-1 diacetate and imaged by confocal microscopy (488 nm). PKC was inhibited in live cells using either Fim-1 diacetate (200 nM) or chelerythrine (200 nM, Sigma) for 2 hours before and during shear stress [38]. PKC was activated in normal glucose cells using phorbol 12-myristate 13-acetate (PMA, 1 µM, Sigma) for 2 hours before and during shear stress [39].

Intracellular ROS was measured using the Image-iT® Live Green Reactive Oxygen Species Detection Kit (Molecular Probes) as per manufacturer instructions. Carboxy-H_2_DCFDA is deacetylated into carboxy-DCFH by intracellular esterases. Intracellular ROS then oxidize carboxy-DCFH to carboxy-DCF, which fluoresces green. 25 µM carboxy-H_2_DCFDA was applied to live cells for 30 minutes, after which cells were washed and imaged by confocal microscopy (488 nm). *N*-acetyl-*L*-cysteine (NAC, Sigma, 10 µg/mL), applied 2 hours before and during shear stress, was used to scavenge ROS.

Endothelial cell VEGF release was measured using a VEGF ELISA (R&D Systems) as per manufacturer instructions. PAEC were exposed to LG, NG, and HG medium for 24–48 hours. Conditioned medium samples were collected and either immediately measured or frozen at −80°C and measured within one week. VEGF activity was blocked in LG cells using a VEGF neutralizing antibody (Millipore, 1 µg/mL). Alternatively, VEGF (Peprotech) was added to NG cells at 100 ng/mL for short-term experiments (<30 minutes) and 50 ng/mL for long-term experiments. Since both glucose and VEGF interfere with cell-cell junctions, β-catenin (Invitrogen) was used to quantify adherens junction integrity. PAEC were fixed in 4% paraformaldehyde, permeabilized with 0.1% Triton, β-catenin and nuclei were labeled with a primary β-catenin antibody (1∶200, Invitrogen) and bisbenzimide (0.2 µg/mL), respectively, and imaged by confocal microscopy.

### Signaling Pathways: FAK and Akt

PI3K is phosphorylated by cell-cell junction mechanosensory complex activation [27]. PI3K activates integrins, leading to FAK phosphorylation and subsequent Rho/Rac pathway activation leading to actin reorganization. PI3K also phosphorylates Akt, which leads to eNOS phosphorylation and nitric oxide release. Both are phosphorylated within seconds of shear stress initiation. We investigated FAK by immunofluorescent microscopy after 30 seconds shear stress to rapidly visualize activation at focal adhesions. We measured Akt phosphorylation, which is immediately downstream of PI3K, after 30 minutes to obtain adequate activated protein quantity for Western blot. For FAK, cells were fixed in paraformaldehyde as described and labeled with a phosphorylated FAK primary antibody (p-FAK, Y397, 1∶100, Cell Signaling). Samples were then incubated with the appropriate secondary antibody (Alexa Fluor® 633; Invitrogen) and bisbenzimide and imaged by confocal microscopy. We measured pFAK by immunofluorescent microscopy to specifically analyze only pFAK localized to focal adhesions.

For Akt phosphorylation, cells in either static conditions or exposed to 30 minutes shear stress were lysed in ice cold lysis buffer (20 mM Tris, 150 mM NaCl, 1% Triton X-100, 2 mM EDTA, 2 mM PMSF, 0.1% SDS, 1 µg/ml leupeptin, 2 mM NaVO_4_, 50 mM mM NaF, 10% glycerol, complete protease inhibitor, pH 7.4). Normalized protein samples were separated by gel electrophoresis, transferred to nitrocellulose membranes, and labeled for p-Akt (Ser473, 1∶1000, Cell Signaling) and Akt (1∶1000, Cell Signaling). Protein bands were detected using an enhanced chemiluminescence kit (Western Lightning, PerkinElmer) and visualized with a Fluorchem digital imager (Alpha Innotech). Band intensity was quantified using ImageJ.

### Statistical Analysis

Matlab’s statistics toolbox was used for all statistical analyses. Data are graphed as mean ± standard deviation. Student’s t- test was used to compare two groups, and n-way ANOVA with a Tukey-Kramer post test was used to compare multiple groups. p<0.01 is indicated with an asterisk (*), and p<0.05 is indicated by a pound sign (#). Matlab was used to quantify immunofluorescence intensity in at least 3 images per sample to create a sample average. Two samples were then analyzed for each experimental condition, and these are the averages and standard deviations shown. Shear stress experiments were performed in duplicate, and each experiment was repeated three times. All other experiments were performed in triplicate and repeated at least two times. For certain experiments, image contrast was consistently enhanced in all samples using the adaptive histogram equalization program (adapthisteq.m) in Matlab.

## Results

### Actin Alignment and eNOS Activation

**Figure 1:**
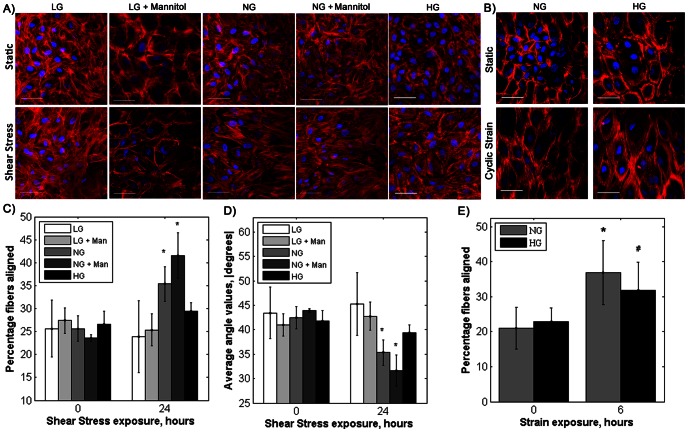
Endothelial cells in low and high glucose medium did not align in the flow direction, whereas cells in normal glucose aligned. Cells in high glucose also aligned in response to cyclic strain. Actin (red) and nuclei (blue) before and after A) shear stress and B) cyclic strain, both applied in the horizontal direction. Scale bar = 50 µm. C) Percentage aligned actin fibers for shear stress, D) average actin fiber angle (absolute value) for shear stress, and E) percentage aligned actin fibers for cyclic strain. *p<0.01, #p<0.05 compared to static sample for the same culture condition. Shear stress experiments were completed in duplicate, and cyclic strain experiments were completed in triplicate. Each experiment was repeated three times.

Endothelial cells are dysfunctional in both low and high glucose environments [8], [17], which may contribute to accelerated atherosclerosis in people with diabetes. Failure of endothelial cells to align to flow correlates with a pro-atherosclerotic phenotype [20]. We therefore measured endothelial cell actin alignment in response to shear stress in altered glucose conditions. Endothelial cells cultured in LG, LG osmotic control and HG medium did not align their actin fibers after 24 hours of 20 dynes/cm^2^ shear stress (Figure 1A). For NG and HG osmotic control cells exposed to flow, aligned actin fibers (−20° to 20°) increased by 10.1% and 18.0% from static conditions, respectively (Figure 1C, p<0.01 by ANOVA). In contrast, LG, LG osmotic control, and HG cells exposed to shear stress showed less than a 3% change in aligned actin fibers compared to static control. These data were confirmed via average actin fiber angle (absolute value, Figure 1D, p<0.01 by ANOVA). Average actin angle decreased 7.3° with shear stress for NG cells and 12.3° for HG osmotic control cells (p<0.01). In contrast, average actin angle for LG, LG osmotic control and HG cells exposed to flow changed by less than 3°.

Interestingly, endothelial cells exposed to cyclic strain did align actin fibers perpendicular to the strain direction for both NG and HG cells. Aligned actin fibers (70° to 110°) increased 15% with 6 hours of cyclic strain for NG cells and 9% for HG cells, both of which were statistically significant changes (Figure 1B, E; p<0.01). There was no difference between the normal and high glucose alignment results.

**Figure 2:**
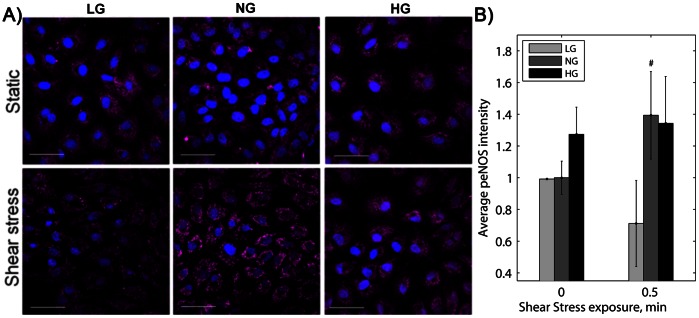
eNOS phosphorylation increased with shear stress for endothelial cells in normal glucose but did not significantly change for cells in low or high glucose. A) p-eNOS (magenta) and nuclei (blue) after 30 seconds shear stress (horizontal direction). Scale bar = 50 µm. B) p-eNOS mean fluorescence intensity. * p<0.01 compared to static sample for the same glucose condition. Experiments were completed in duplicate and repeated three times.

Endothelial cells release NO as both a vasodilatory and anti-inflammatory molecule in response to shear stress, and decreased NO release is part of the atheroprone endothelial cell phenotype. eNOS phosphorylation is required for NO production, and in our previous work, eNOS phosphorylation translated to NO release. NO is difficult to measure in a flow apparatus due to the large fluid volume. We therefore compared phosphorylated eNOS (p-eNOS) in LG, NG, and HG cells before and after shear stress. For NG cells, p-eNOS intensity increased nearly 40% following 30 seconds of 20 dynes/cm^2^ shear stress. Neither LG nor HG cells showed a significant increase in p-eNOS after shear stress compared to static conditions (Figure 2, p<0.01 by ANOVA). While p-eNOS was elevated in HG cells cultured following shear stress, p-eNOS was similarly elevated in these cells in static conditions, and thus there was no significant change with shear stress.

### FAK and Akt Activation

**Figure 3:**
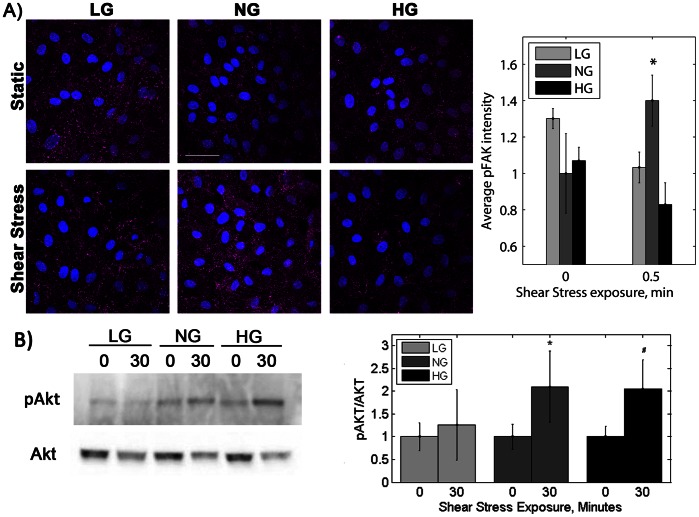
PAEC in low and high glucose did not activate FAK in response to shear stress, whereas shear stress did increase phosphorylated Akt in high glucose cells. A) p-FAK (magenta) and nuclei (blue) after 30 seconds shear stress (horizontal direction). Scale bar = 50 µm. p-FAK mean fluorescence intensity. *p<0.01 compared to static sample for the same glucose condition. B) Akt phosphorylation after 30 minutes of shear stress. p-Akt normalized to total Akt. *p<0.01, ^#^p<0.05 compared to static sample for the same glucose condition. Experiments were completed in duplicate and repeated three times.

We hypothesized that endothelial cells in altered glucose did not align or phosphorylate eNOS in response to shear stress due to disruption of the adherens junction mechanosensory complex. VEGFR2 in this complex activates PI3K, which then activates integrins in focal adhesions. FAK, which is phosphorylated within minutes of focal adhesion activation, then signals towards actin alignment. We visualized p-FAK localized to focal adhesions by immunofluorescent microscopy to determine if shear stress-induced integrin activation was changed in altered glucose conditions. Shear stress did not activate FAK in endothelial cells grown in low and high glucose medium. p-FAK (Y397) fluorescence intensity increased by 40% in NG cells after 30 seconds shear stress (Figure 3A, p<0.01 by ANOVA). However, in both LG and HG cells, there was no significant increase in p-FAK after shear stress. p-FAK was slightly elevated in static LG cells compared to cells in normal and high glucose medium.

PI3K activated by the adherens junction mechanosensory complex also phosphorylates Akt, which then phosphorylates eNOS to stimulate NO release [27], [30]. In our experiments, Akt was activated by shear stress for cells cultured in normal and high but not low glucose. Both NG and HG cells showed two fold p-Akt increase with 30 minutes shear stress (Figure 3B), whereas LG cells did not show a significant p-Akt increase. These data suggest that the mechanisms by which low and high glucose alter endothelial cell shear stress response are different. Low glucose appears to interfere with mechanotransduction upstream of PI3K, whereas high glucose interferes either downstream of PI3K or along a parallel mechanotransduction pathway (e.g., integrins).

### ROS and PKC Levels

**Figure 4:**
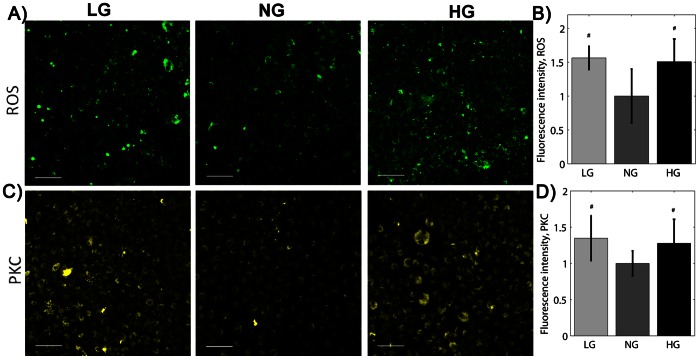
ROS and PKC were elevated in both low and high glucose. A) carboxy-H_2_DCFDA (ROS, green) and C) Fim-1 diacetate (PKC, yellow) after 48 hours in different glucose conditions. Scale bar = 50 µm. B) ROS and D) PKC fluorescence mean fluorescence intensity. ^#^p<0.05 compared to NG cells. Experiments were completed in triplicate and repeated three times.

We next measured ROS and PKC levels, since both these factors contribute to endothelial cell dysfunction in altered glucose [40]. PAEC exposed to two days of LG or HG medium showed elevated ROS compared to the NG control. Carboxy-DCF fluorescence intensity was 50.6% higher for HG cells and 56.3% higher for LG cells compared to NG cells (Figure 4A, B, p<0.01 by ANOVA). Similarly, PKC in cells in low and high glucose was elevated compared to cells cultured in normal glucose. Fim-1 diacetate fluorescent intensity was 34.8% and 27.7% higher in LG and HG cells, respectively, than in NG cells (Figure 4C, D, p<0.01 by ANOVA). ROS and PKC were similarly elevated in PAEC exposed to LG or HG medium under fluid flow conditions.

### High Glucose: PKC Effect on FAK Activation and Actin Alignment

**Figure 5:**
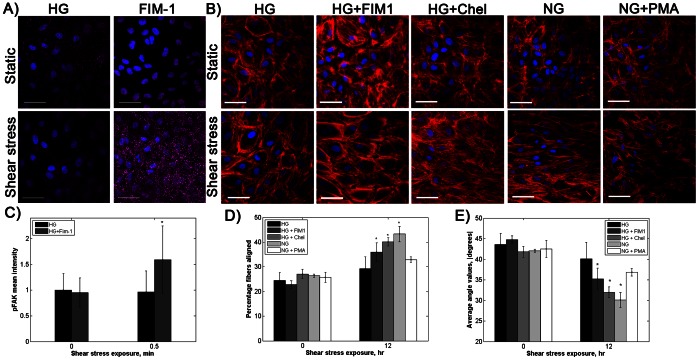
PKC blockade restored FAK phosphorylation and actin alignment in response to shear stress in cells cultured in high glucose. A) p-FAK (magenta) and nuclei (blue) with 2 hours Fim-1 diacetate (200 nM) followed by 30 seconds shear stress (horizontal direction). Scale bar = 50 µm. C) p-FAK mean fluorescence intensity. * p<0.01 compared to static. B) Actin (red) and nuclei (blue) for high glucose cells with PKC blocked by 200nM Fim-1 diacetate and 200 nM chelerythrine, and normal glucose cells with PKC activated by 1 µM PMA. Shear stress was applied for 12 hours (horizontal direction). Scale bar = 50 µm. D) and E) Actin aligned fiber percentage and average angle absolute value. * p<0.01 compared to static sample for the same culture condition. Experiments were completed in duplicate and repeated three times.

PKC is important in shear stress signaling and FAK activation [41]–[43]. However, glucose-induced PKC elevation in mouse renal mesangial cells interfered with FAK activation and cell contractility [44]. We blocked PKC in cells cultured in both low and high glucose medium using Fim-1 diacetate or chelerythrine and measured FAK activation and actin alignment. PKC blockade increased FAK phosphorylation and actin alignment in response to shear stress in HG but not LG cells. HG cells treated with Fim-1 diacetate for 2 hours prior to and during shear stress exposure had a 60% increase in p-FAK intensity compared to static conditions, whereas untreated HG cells showed no significant FAK activation (Figure 5A, C). Similarly, blocking PKC enabled actin fiber alignment after 12 hours shear stress in HG cells (Figure 5B, D, E, p<0.01 by ANOVA) and reduced elevated basal p-eNOS levels in HG cells. Aligned fiber percentage increased by 8% and mean actin fiber angle decreased by 6° with shear stress for HG cells treated with either PKC blocker. The PKC activator PMA also prevented actin fiber alignment in NG cells. Actin fiber alignment for LG cells exposed to shear stress did not change with PKC blockade (data not shown). These results suggest that high glucose impairs endothelial cell actin alignment in response to shear stress via PKC.

### Low Glucose: VEGF Effect on Adherens Junctions and Mechanotransduction

**Figure 6:**
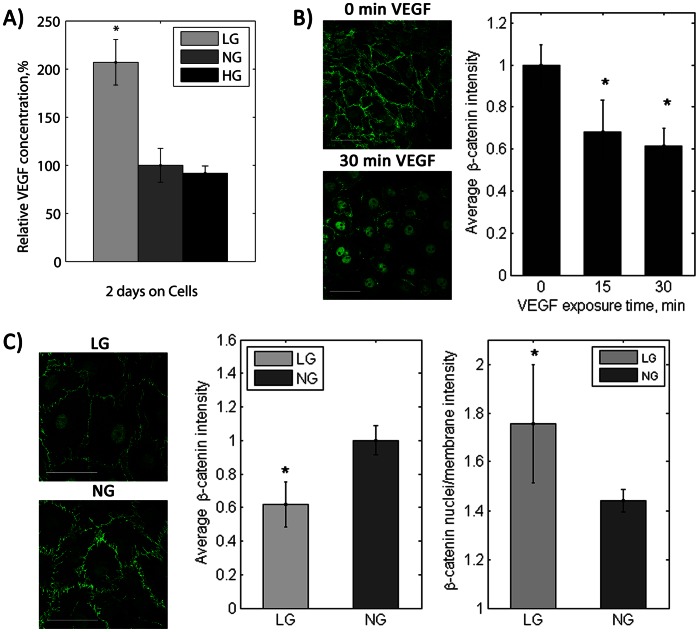
VEGF secreted by endothelial cells in low glucose translocated β-catenin at cell-cell junctions to the nucleus. A) VEGF in conditioned medium after two days. * p<0.01 compared to NG cells. B) β-catenin (green) after VEGF treatment (100 ng/mL) of PAEC in normal glucose.* p<0.01 compared to 0 minutes VEGF exposure. C) β-catenin (green) in PAEC in low glucose. Scale bar = 50 µm. * p<0.01 compared to NG cells. Experiments were completed in triplicate and repeated two times.

Hypoglycemia inhibited both FAK and Akt activation, and several cell types reportedly release VEGF, a permeability factor that disrupts adherens junctions, in response to low glucose [45]–[47]. We therefore examined VEGF release in LG cells and its effect on endothelial cell actin alignment in response to shear stress. Cells cultured in low glucose for two days released twice as much VEGF compared to cells in normal or high glucose (Figure 6A, p<0.01 by ANOVA), and low glucose elevated VEGF release by 24 hours (data not shown). NG cells treated with exogenous VEGF (100 ng/mL for 15 and 30 minutes) showed reduced β-catenin at cell borders (Figure 6B, p<0.01 by ANOVA). Similarly, low glucose decreased overall β-catenin and increased nuclear:membrane β-catenin ratio (Figure 6C). A neutralizing VEGF antibody restored β-catenin at the cell membrane in LG cells (data not shown). These results suggest that VEGF release by cells in low glucose translocates β-catenin from the membrane to the nuclei, thereby disrupting adherens junctions and inhibiting mechanosensory complex formation.

**Figure 7:**
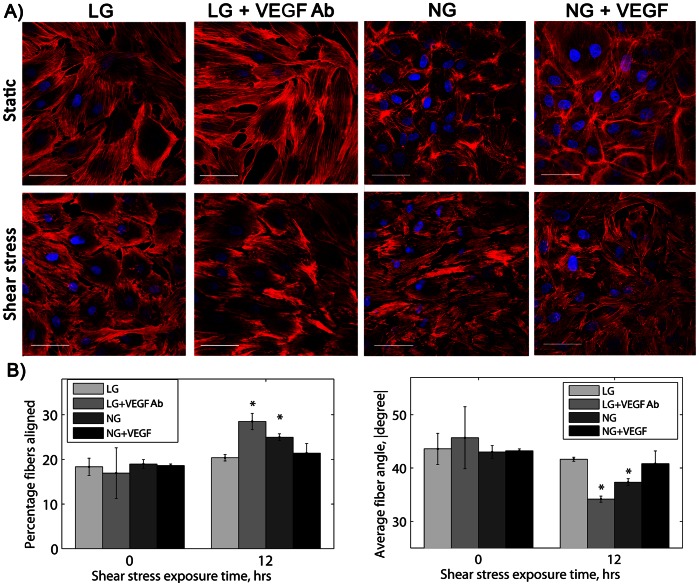
Actin fibers aligned in low glucose with shear stress when VEGF was blocked, and VEGF addition to cells in normal glucose prevented actin alignment with shear stress. A) Actin (red) and nuclei (blue) in low glucose cells with VEGF blocked for 24 hours with a neutralizing antibody (1 µg/ml) or normal glucose cells with VEGF added (50 ng/mL) and exposed to 12 hours shear stress. Scale bar = 50 µm. B) Actin fiber percentage and average actin fiber angle (absolute value). *p<0.01 compared to static sample for the same culture condition. Experiments were completed in duplicate and repeated three times.

To determine if VEGF prevents endothelial cell actin alignment in response to shear stress, we blocked VEGF in LG cells and added VEGF to NG cells. A VEGF neutralizing antibody re-established endothelial cell actin alignment in response to shear stress in low glucose culture, and adding VEGF to the medium prevented actin alignment in cells grown in normal glucose medium (Figure 7A). Aligned actin fiber percentage increased by 11.3% from the static condition for LG cells with the VEGF neutralizing antibody, and average actin fiber angle decreased by 11.5° (Figure 7B, p<0.01 by ANOVA). When 50 ng/mL VEGF was added to NG cells for 24 hours prior to shear stress exposure, neither aligned fiber percentage nor average actin fiber angle changed with flow.

## Discussion

**Figure 8:**
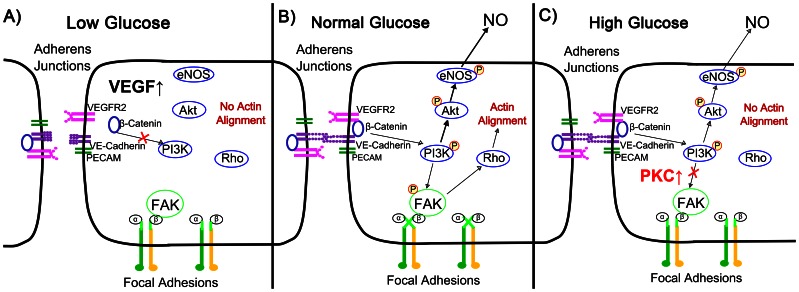
Proposed signaling pathways in low and high glucose. A) Endothelial cells in low glucose release VEGF, which disrupts β-catenin at adherens junctions. This then impairs mechanosensory complex activation due to shear stress, inhibiting downstream responses. B) Cells in normal glucose sense shear stress at the mechanosensory complex, phosphorylate eNOS, and align actin. C) Cells in high glucose maintain an intact mechanosensory complex, however elevated PKC inhibits FAK activation and subsequent actin alignment.

People with diabetes develop early and accelerated atherosclerosis, and disease severity correlates with poor glucose control. While altered glucose is known to contribute to endothelial cell dysfunction, its effect on endothelial cell mechanotransduction had not yet been fully examined. We now present two different mechanisms for impaired mechanotransduction in altered glucose conditions (Figure 8). For endothelial cells in normal glucose, shear stress activates the PECAM/VE-cadherin/VEGFR2 mechanosensory complex, which then phosphorylates PI3K. PI3K in turn activates integrins at focal adhesions (leading to FAK phosphorylation, Rho-GTPase signaling, and actin alignment) and Akt (leading to eNOS phosphorylation and NO release). Hyperglycemia elevates PKC, which impairs FAK phosphorylation and hence endothelial cell actin alignment in response to shear stress (Figure 8C). Hypoglycemia also diminishes endothelial cell shear stress response but through a different mechanism. Cells in low glucose release VEGF, which disrupts cell-cell adherens junctions and thereby prevents mechanosensory complex signaling (Figure 8A). This research advances our understanding of how blood glucose fluctuations in diabetes affect with vascular mechanotransduction and may contribute to a pro-atherosclerotic endothelial phenotype.

Endothelial cell actin alignment remained intact when cells were exposed to cyclic strain in hyperglycemia. This suggests that glucose interrupts mechanotransduction pathways specific to shear stress. Many signaling pathways, including focal adhesion activation via FAK, are common to both shear stress and strain. For example, we previously showed that glycated collagen disrupts endothelial cell response to both shear stress and strain through diminished FAK activation [34], [36]. However, other studies show mechanotransduction differences in shear stress and cyclic strain [48]. The adherens junction mechanosensory complex has to date only been proven important in shear stress response [25]–[27]. Both non-confluent human aortic endothelial cells with FAK silenced by shRNA and FAK null mouse embryonic fibroblasts still aligned perpendicular to the cyclic strain direction, suggesting that FAK may not be necessary for strain mechanotransduction in all cell types and conditions [49], [50].

Both ROS and PKC were elevated in cells in high glucose, yet only PKC blockade restored both FAK phosphorylation and actin alignment. An ROS scavenger applied 2 hours prior to shear stress did not restore FAK activation in high glucose cells. When the same ROS scavenger was kept in the medium during 12 hours of shear stress, actin alignment was restored (data not shown). These data suggest that longer term ROS inhibition may be required to restore endothelial cell shear stress response, or that PKC is a primary mediator for high glucose inhibition of endothelial mechanotransduction in high glucose, although more studies are needed. Interestingly, both ROS and PKC play important roles in endothelial cell shear stress response. 15 minutes of shear stress exposure increased ROS in HUVEC and mediated *c-fos* gene transcription [51]. PKC increased particularly in the cell cortex in response to shear stress [52] and was required for shear stress-induced mitogen-activated protein (MAP) kinase activation in bovine aortic endothelial cells [43] and endothelin-1 release in human umbilical vein endothelial cells exposed to low shear stress levels [42]. Chronically elevated ROS and PKC may be detrimental to cell signaling pathways that are dependent on these molecules, as evidenced by a study showing that glucose-induced elevated PKC reduced PKC-dependent mesangial cell contractility [44]. Alternatively, reducing global ROS and PKC levels may restore endothelial cell alignment but inhibit other critical shear stress responses.

In low glucose, endothelial cell mechanotransduction is disrupted through cell compensatory pathways. In both hypoxia and hypoglycemia, cells release VEGF to bring oxygen and glucose to the starved tissue via increased vascular permeability and microvascular angiogenesis [45], [53]–[55]. VEGF-induced permeability is mediated through adherens junction disruption via β-catenin, which together with α-catenin links VE-cadherin to the actin cytoskeleton [46]. VEGF stimulation leads to β-catenin phosphorylation, which dissociates β-catenin from VE-cadherin and moves it to the cytosol or nucleus [55]. β-catenin has been shown to associate with VE-cadherin, VEGFR2, and PECAM through immunoprecipitation and knockout models [56], [57]. VE-cadherin and β-catenin are required for adherens junction mechanosensory complex assembly and subsequent signaling, since neither VE-cadherin^−/−^ nor β-catenin^−/−^ endothelial cells activated integrins in response to flow [27]. In low glucose, VEGF release and cell stimulation moves β-catenin away from the cell membrane, which prevents mechanotransduction signaling from this complex. VEGF may also cause the elevated ROS and PKC levels in low glucose. When VEGF binds to VEGFR2, the small GTPase Rac1 is activated. Rac1 stimulates NADPH oxidase at the plasma membrane to produce ROS [58]. VEGF-induced ROS via Rac1 increase endothelial permeability and activate β-catenin and VE-cadherin [59]. VEGF also enhances PKC activation in a time dependent manner through a PI3K and PLCγ mediated pathway [60].

These studies show that both high and low glucose promote an atheroprone endothelial cell phenotype in response to the onset of laminar shear stress. However, this research has similar limited applicability to human atherosclerosis as many *in vitro* shear stress studies. Atherosclerosis in humans develops over years, but the specific cell signaling events occur on much shorter time scales that are similar to those in our experiments. For example, endothelial cell dysfunction can be transiently induced by inflammation, elevated glucose, or hypercholesterolemia. This then leads to short term changes in cell permeability and inflammatory cell recruitment to the atherosclerotic site, which causes the plaque to grow. Over years of these short term insults, a large atherosclerotic plaque develops. Thus short term effects are relevant to long term atherosclerotic plaque development. Atherosclerosis also develops in locations of disturbed rather than laminar flow, although some suggest that the disease is more diffuse in people with diabetes. Endothelial cells exposed to disturbed flow do not align, and it would therefore not be possible to measure an effect of glucose on cell alignment in these conditions. However, it may be interesting to determine if glucose exacerbates other aspects of the atheroprone phenotype in disturbed flow, such as inflammatory adhesion molecule expression.

While our research shows an important glucose effect on endothelial cell mechanotransduction, this study has several limitations. In our research, isolated endothelial cells on glass slides were exposed to constant laminar shear stress using an *in vitro* system. *In vivo,* endothelial cells grow on the more compliant vascular wall, interact with neighboring cells such as smooth muscle cells, and experience pulsatile flow. In the PKC, ROS, and FAK experiments, we used immunofluorescence to visualize changes in low and high glucose. While we are confident in our results, since they were repeatable and supported by our chemical inhibition studies, these methods are only semi-quantitative. Additional measurement methods and studies further delineating these pathways would confirm these results. The glucose levels we used are at the extreme of both low and high glucose levels experienced by diabetic patients. We used these glucose extremes to enhance changes observed in short term *in vitro* culture. Humans and animals with diabetes also have many other biochemical changes in addition to altered blood glucose, including elevated advanced glycation end products and altered insulin levels. While we hope in the future to confirm our *in vitro* data using *in vivo* human or animal data, *in vivo* studies are challenging due to the complexities of both the diabetic biochemical environment and the vascular mechanical environment. In our case, the simplified experimental conditions allowed us to isolate and identify the mechanisms by which glucose affects endothelial response to shear stress.

High glucose has long been considered detrimental to the vasculature, however increasingly low glucose is also implicated in cardiovascular disease. We now show that elevated PKC in high glucose and VEGF release in low glucose prevent endothelial cell alignment in response to shear stress. This research clarifies endothelial cell response to physiologic flow at both ends of the glucose spectrum. By understanding these mechanisms, we hope to identify targets and develop treatments and preventative measures to protect the cardiovascular system from the effects of diabetes.

## References

[1] Centers for Disease Control and Prevention (2011) National diabetes fact sheet: national estimates and general information on diabetes and prediabetes in the United States, 2011. Atlanta, GA: U.S. Department of Health and Human Services, Centers for Disease Control and Prevention.

[2] Go AS, Mozaffarian D, Roger VL, Benjamin EJ, Berry JD, et al (2013) Heart Disease and Stroke Statistics–2013 Update: A Report From the American Heart Association. Circulation 127: e6–e245. 23239837 10.1161/CIR.0b013e31828124ad PMC5408511

[3] Nicholls SJ, Tuzcu EM, Kalidindi S, Wolski K, Moon K-W, et al (2008) Effect of Diabetes on Progression of Coronary Atherosclerosis and Arterial Remodeling: A Pooled Analysis of 5 Intravascular Ultrasound Trials. Journal of the American College of Cardiology 52: 255–262. 18634979 10.1016/j.jacc.2008.03.051

[4] Wagenknecht LE, Zaccaro D, Espeland MA, Karter AJ, O’Leary DH, et al (2003) Diabetes and Progression of Carotid Atherosclerosis. Arteriosclerosis, thrombosis, and vascular biology 23: 1035–1041. 10.1161/01.ATV.0000072273.67342.6D 12702517

[5] Molnar GD, Taylor WF, Langworthy A (1974) On measuring the adequacy of diabetes regulation: Comparison of continuously monitored blood glucose patterns with values at selected time points. Diabetologia 10: 139–143. 4844189 10.1007/BF01219670

[6] Nathan D, Cleary P, Backlund J, Genuth S, Lachin J, et al (2005) Intensive diabetes treatment and cardiovascular disease in patients with type 1 diabetes. New England Journal of Medicine 353: 2643–2653. 16371630 10.1056/NEJMoa052187 PMC2637991

[7] Michiels C (2003) Endothelial Cell Function. Journal of Cellular Physiology 196: 430–443. 12891700 10.1002/jcp.10333

[8] Hempel A, Maasch C, Heintze U, Lindschau C, Dietz R, et al (1997) High Glucose Concentrations Increase Endothelial Cell Permeability via Activation of Protein Kinase Cα. Circulation Research 81: 363–371. 9285638 10.1161/01.res.81.3.363

[9] Richardson M, Hadcock S, DeReske M, Cybulsky M (1994) Increased expression in vivo of VCAM-1 and E-selectin by the aortic endothelium of normolipemic and hyperlipemic diabetic rabbits. Arteriosclerosis, thrombosis, and vascular biology 14: 760–769. 10.1161/01.atv.14.5.760 7513552

[10] Chakravarthy U, Hayes RG, Stitt AW, McAuley E, Archer DB (1998) Constitutive nitric oxide synthase expression in retinal vascular endothelial cells is suppressed by high glucose and advanced glycation end products. Diabetes 47: 945–952. 9604873 10.2337/diabetes.47.6.945

[11] Hamuro M, Polan J, Natarajan M, Mohan S (2002) High glucose induced nuclear factor kappa B mediated inhibition of endothelial cell migration. Atherosclerosis 162: 277–287. 11996947 10.1016/s0021-9150(01)00719-5

[12] Lorenzi M, Cagliero E, Toledo S (1985) Glucose toxicity for human endothelial cells in culture. Delayed replication, disturbed cell cycle, and accelerated death. Diabetes 34: 621–627. 3924693 10.2337/diab.34.7.621

[13] Martin A, Komada MR, Sane DC (2003) Abnormal angiogenesis in diabetes mellitus. Medicinal Research Reviews 23: 117–145. 12500286 10.1002/med.10024

[14] Du X-L, Edelstein D, Rossetti L, Fantus IG, Goldberg H, et al (2000) Hyperglycemia-induced mitochondrial superoxide overproduction activates the hexosamine pathway and induces plasminogen activator inhibitor-1 expression by increasing Sp1 glycosylation. Proceedings of the National Academy of Sciences 97: 12222–12226. 10.1073/pnas.97.22.12222 PMC17322 11050244

[15] Brownlee M (1995) Advanced protein glycosylation in diabetes and aging. Annual Review of Medicine 46: 223–234. 10.1146/annurev.med.46.1.223 7598459

[16] Lee TS, Saltsman KA, Ohashi H, King GL (1989) Activation of protein kinase C by elevation of glucose concentration: proposal for a mechanism in the development of diabetic vascular complications. Proceedings of the National Academy of Sciences 86: 5141–5145. 10.1073/pnas.86.13.5141 PMC297573 2740348

[17] Wang J, Alexanian A, Ying R, Kizhakekuttu TJ, Dharmashankar K, et al (2012) Acute Exposure to Low Glucose Rapidly Induces Endothelial Dysfunction and Mitochondrial Oxidative Stress. Arteriosclerosis, thrombosis, and vascular biology 32: 712–720. 10.1161/ATVBAHA.111.227389 PMC3319449 22207730

[18] Dewey C, Bussolari S, Gimbrone M, Davies PF (1981) The dynamic response of vascular endothelial cells to fluid shear stress. Journal of Biomechanical Engineering 103: 177–185. 7278196 10.1115/1.3138276

[19] Fisslthaler B, Dimmeler S, Hermann C, Busse R, Fleming I (2000) Phosphorylation and activation of the endothelial nitric oxide synthase by fluid shear stress. Acta Physiologica Scandinavica 168: 81–88. 10691783 10.1046/j.1365-201x.2000.00627.x

[20] Hahn C, Schwartz MA (2009) Mechanotransduction in vascular physiology and atherogenesis. Nature Reviews Molecular Cell Biology 10: 53–62. 19197332 10.1038/nrm2596 PMC2719300

[21] Nicholls SJ, Tuzcu EM, Crowe T, Sipahi I, Schoenhagen P, et al (2006) Relationship between cardiovascular risk factors and atherosclerotic disease burden measured by intravascular ultrasound. J Am Coll Cardiol 47: 1967–1975. 16697312 10.1016/j.jacc.2005.12.058

[22] Vigorita VJ, Moore GW, Hutchins GM (1980) Absence of correlation between coronary arterial atherosclerosis and severity or duration of diabetes mellitus of adult onset. Am J Cardiol 46: 535–542. 7416013 10.1016/0002-9149(80)90500-7

[23] Davies PF (1995) Flow-mediated endothelial mechanotransduction. Physiological Reviews 75: 519–560. 7624393 10.1152/physrev.1995.75.3.519 PMC3053532

[24] Morss AS, Edelman ER (2007) Glucose modulates basement membrane fibroblast growth factor-2 via alterations in endothelial cell permeability. J Biol Chem 282: 14635–14644. 17327226 10.1074/jbc.M608565200

[25] Osawa M, Masuda M, Kusano K-i, Fujiwara K (2002) Evidence for a role of platelet endothelial cell adhesion molecule-1 in endothelial cell mechanosignal transduction. The Journal of Cell Biology 158: 773–785. 12177047 10.1083/jcb.200205049 PMC2174013

[26] Shay-Salit A, Shushy M, Wolfovitz E, Yahav H, Breviario F, et al (2002) VEGF receptor 2 and the adherens junction as a mechanical transducer in vascular endothelial cells. Proc Natl Acad Sci U S A 99: 9462–9467. 12080144 10.1073/pnas.142224299 PMC123163

[27] Tzima E, Irani-Tehrani M, Kiosses WB, Dejana E, Schultz DA, et al (2005) A mechanosensory complex that mediates the endothelial cell response to fluid shear stress. Nature 437: 426–431. 16163360 10.1038/nature03952

[28] Tzima E, del Pozo MA, Shattil SJ, Chien S, Schwartz MA (2001) Activation of integrins in endothelial cells by fluid shear stress mediates Rho-dependent cytoskeletal alignment. The EMBO Journal 20: 4639–4647. 11532928 10.1093/emboj/20.17.4639 PMC125600

[29] Ridley AJ, Hall A (1992) The small GTP-binding protein rho regulates the assembly of focal adhesions and actin stress fibers in response to growth factors. Cell 70: 389–399. 1643657 10.1016/0092-8674(92)90163-7

[30] Dimmeler S, Fleming I, Fisslthaler B, Hermann C, Busse R, et al (1999) Activation of nitric oxide synthase in endothelial cells by Akt-dependent phosphorylation. Nature 399: 601–605. 10376603 10.1038/21224

[31] Brower J, Targovnik J, Bowen B, Caplan M, Massia S (2009) Elevated Glucose Impairs the Endothelial Cell Response to Shear Stress. Cellular and Molecular Bioengineering 2: 533–543.

[32] Brower JB, Targovnik JH, Caplan MR, Massia SP (2010) High glucose-mediated loss of cell surface heparan sulfate proteoglycan impairs the endothelial shear stress response. Cytoskeleton 67: 135–141. 20217676 10.1002/cm.20430

[33] Tarbell JM, Pahakis MY (2006) Mechanotransduction and the glycocalyx. Journal of Internal Medicine 259: 339–350. 16594902 10.1111/j.1365-2796.2006.01620.x

[34] Kemeny SF, Figueroa DS, Andrews AM, Barbee KA, Clyne AM (2011) Glycated collagen alters endothelial cell actin alignment and nitric oxide release in response to fluid shear stress. Journal of Biomechanics 44: 1927–1935. 21555127 10.1016/j.jbiomech.2011.04.026

[35] Malek AM, Alper SL, Izumo S (1999) Hemodynamic Shear Stress and Its Role in Atherosclerosis. JAMA 282: 2035–2042. 10591386 10.1001/jama.282.21.2035

[36] Figueroa D, Kemeny S, Clyne A (2011) Glycated Collagen Impairs Endothelial Cell Response to Cyclic Stretch. Cellular and Molecular Bioengineering 4: 220–230.

[37] Kemeny SF, Clyne AM (2011) A Simplified Implementation of Edge Detection in MATLAB is Faster and More Sensitive than Fast Fourier Transform for Actin Fiber Alignment Quantification. Microscopy and Microanalysis 17: 156–166. 21385521 10.1017/S143192761100002X

[38] Herbert JM, Augereau JM, Gleye J, Maffrand JP (1990) Chelerythrine is a potent and specific inhibitor of protein kinase C. Biochem Biophys Res Commun. 172: 993–999. 10.1016/0006-291x(90)91544-3 2244923

[39] Niedel JE, Kuhn LJ, Vandenbark GR (1983) Phorbol diester receptor copurifies with protein kinase C. Proc Natl Acad Sci U S A. 80: 36–40. 10.1073/pnas.80.1.36 PMC393304 6296873

[40] Brownlee M (2001) Biochemistry and molecular cell biology of diabetic complications. Nature 414: 813–820. 11742414 10.1038/414813a

[41] Lewis JM, Cheresh DA, Schwartz MA (1996) Protein kinase C regulates alpha v beta 5-dependent cytoskeletal associations and focal adhesion kinase phosphorylation. Journal of Cell Biology 134: 1323–1332. 8794871 10.1083/jcb.134.5.1323 PMC2120976

[42] Kuchan MJ, Frangos JA (1993) Shear stress regulates endothelin-1 release via protein kinase C and cGMP in cultured endothelial cells. American Journal of Physiology - Heart and Circulatory Physiology 264: H150–H156. 10.1152/ajpheart.1993.264.1.H150 8381608

[43] Tseng H, Peterson TE, Berk BC (1995) Fluid Shear Stress Stimulates Mitogen-Activated Protein Kinase in Endothelial Cells. Circulation Research 77: 869–878. 7554140 10.1161/01.res.77.5.869

[44] Chen J-S, Lee H-S, Jin J-S, Chen A, Lin S-H, et al (2004) Attenuation of mouse mesangial cell contractility by high glucose and mannitol: Involvement of protein kinase C and focal adhesion kinase. Journal of Biomedical Science 11: 142–151. 14966364 10.1007/BF02256557

[45] Shweiki D, Neeman M, Itin A, Keshet E (1995) Induction of vascular endothelial growth factor expression by hypoxia and by glucose deficiency in multicell spheroids: implications for tumor angiogenesis. Proceedings of the National Academy of Sciences 92: 768–772. 10.1073/pnas.92.3.768 PMC42701 7531342

[46] Dejana E, Bazzoni G, Lampugnani MG (1999) Vascular endothelial (VE)-cadherin: Only an intercellular glue? Experimental Cell Research 252: 13–19. 10502395 10.1006/excr.1999.4601

[47] Esser S, Lampugnani MG, Corada M, Dejana E, Risau W (1998) Vascular endothelial growth factor induces VE-cadherin tyrosine phosphorylation in endothelial cells. Journal of Cell Science 111: 1853–1865. 9625748 10.1242/jcs.111.13.1853

[48] Shikata Y, Rios A, Kawkitinarong K, DePaola N, Garcia JGN, et al (2005) Differential effects of shear stress and cyclic stretch on focal adhesion remodeling, site-specific FAK phosphorylation, and small GTPases in human lung endothelial cells. Experimental Cell Research 304: 40–49. 15707572 10.1016/j.yexcr.2004.11.001

[49] Hsu H-J, Lee C-F, Locke A, Vanderzyl SQ, Kaunas R (2010) Stretch-Induced Stress Fiber Remodeling and the Activations of JNK and ERK Depend on Mechanical Strain Rate, but Not FAK. PLoS ONE 5. 10.1371/journal.pone.0012470 PMC2930005 20814573

[50] Ngu H, Feng Y, Lu L, Oswald SJ, Longmore GD, et al (2010) Effect of focal adhesion proteins on endothelial cell adhesion, motility and orientation response to cyclic strain. Ann Biomed Eng 38: 208–222. 19856213 10.1007/s10439-009-9826-7

[51] Hsieh H-J, Cheng C-C, Wu S-T, Chiu J-J, Wung B-S, et al (1998) Increase of reactive oxygen species (ROS) in endothelial cells by shear flow and involvement of ROS in shear-induced c-fos expression. Journal of Cellular Physiology 175: 156–162. 9525474 10.1002/(SICI)1097-4652(199805)175:2<156::AID-JCP5>3.0.CO;2-N

[52] Hu YL, Chien S (1997) Effects of shear stress on protein kinase C distribution in endothelial cells. J Histochem Cytochem 45: 237–249. 9016313 10.1177/002215549704500209

[53] Forsythe JA, Jiang BH, Iyer NV, Agani F, Leung SW, et al (1996) Activation of vascular endothelial growth factor gene transcription by hypoxia-inducible factor 1. Molecular and Cellular Biology 16: 4604–4613. 8756616 10.1128/mcb.16.9.4604 PMC231459

[54] Ferrara N, Davis-Smyth T (1997) The Biology of Vascular Endothelial Growth Factor. Endocrine Reviews 18: 4–25. 9034784 10.1210/edrv.18.1.0287

[55] Esser S, Lampugnani MG, Corada M, Dejana E, Risau W (1998) Vascular endothelial growth factor induces VE-cadherin tyrosine phosphorylation in endothelial cells. Journal of Cell Science 111: 1853–1865. 9625748 10.1242/jcs.111.13.1853

[56] Carmeliet P, Lampugnani MG, Moons L, Breviario F, Compernolle V, et al (1999) Targeted deficiency or cytosolic truncation of the VE-cadherin gene in mice impairs VEGF-mediated endothelial survival and angiogenesis. Cell 98: 147–157. 10428027 10.1016/s0092-8674(00)81010-7

[57] Biswas P, Canosa S, Schoenfeld J, Schoenfeld D, Tucker A, et al (2003) PECAM-1 promotes β-catenin accumulation and stimulates endothelial cell proliferation. Biochemical and Biophysical Research Communications 303: 212–218. 12646189 10.1016/s0006-291x(03)00313-9

[58] Abid MR, Tsai JC, Spokes KC, Deshpande SS, Irani K, et al (2001) Vascular endothelial growth factor induces manganese-superoxide dismutase expression in endothelial cells by a Rac1-regulated NADPH oxidase-dependent mechanism. The FASEB Journal 15: 2548–2550. 11641265 10.1096/fj.01-0338fje

[59] Monaghan-Benson E, Burridge K (2009) The Regulation of Vascular Endothelial Growth Factor-induced Microvascular Permeability Requires Rac and Reactive Oxygen Species. Journal of Biological Chemistry 284: 25602–25611. 19633358 10.1074/jbc.M109.009894 PMC2757962

[60] Xia P, Aiello LP, Ishii H, Jiang ZY, Park DJ, et al (1996) Characterization of Vascular Endothelial Growth Factor’s Effect on the Activation of Protein Kinase C, Its Isoforms, and Endothelial Cell Growth. The Journal of clinical Investigation 98: 2018–2026. 8903320 10.1172/JCI119006 PMC507645

